# Effects of Ezetimibe/Simvastatin and Rosuvastatin on Oxidative Stress in Diabetic Neuropathy: A Randomized, Double-Blind, Placebo-Controlled Clinical Trial

**DOI:** 10.1155/2015/756294

**Published:** 2015-07-28

**Authors:** Geannyne Villegas-Rivera, Luis Miguel Román-Pintos, Ernesto Germán Cardona-Muñoz, Oscar Arias-Carvajal, Adolfo Daniel Rodríguez-Carrizalez, Rogelio Troyo-Sanromán, Fermín Paul Pacheco-Moisés, Aldo Moreno-Ulloa, Alejandra Guillermina Miranda-Díaz

**Affiliations:** ^1^Instituto de Investigación Clínica de Occidente, 45030 Guadalajara, JAL, Mexico; ^2^Instituto de Terapéutica Experimental y Clínica, Departamento de Fisiología, Centro Universitario de Ciencias de la Salud, Universidad de Guadalajara, 44340 Guadalajara, JAL, Mexico; ^3^Departamento de Farmacobiología, Centro Universitario de Ciencias Exactas e Ingenierías, Universidad de Guadalajara, 44100 Guadalajara, JAL, Mexico; ^4^Sección de Estudios de Posgrado, Escuela Superior de Medicina, Instituto Politécnico Nacional, 11340 Ciudad de México, DF, Mexico; ^5^Department of Medicine, University of California, San Diego, CA 92093, USA

## Abstract

*Objective*. To evaluate the effects of ezetimibe/simvastatin (EZE/SIMV) and rosuvastatin (ROSUV) on oxidative stress (OS) markers in patients with diabetic polyneuropathy (DPN). *Methods*. We performed a randomized, double-blind, placebo-controlled phase III clinical trial in adult patients with Type 2 Diabetes Mellitus (T2DM) and DPN, as evaluated by composite scores and nerve conduction studies (NCS). Seventy-four subjects with T2DM were allocated 1 : 1 : 1 to placebo, EZE/SIMV 10/20 mg, or ROSUV 20 mg for 16 weeks. All patients were assessed before and after treatment: primary outcomes were lipid peroxidation (LPO), and nitric oxide (NO) surrogate levels in plasma; secondary outcomes included NCS, neuropathic symptom scores, and metabolic parameters. Data were expressed as mean ± SD or SEM, frequencies, and percentages; we used nonparametric analysis. *Results*. LPO levels were reduced in both statin arms after 16 weeks of treatment (*p* < 0.05 versus baseline), without changes in the placebo group. NO levels were not significantly affected by statin treatment, although a trend towards significance concerning increased NO levels was noted in both statin arms. No significant changes were observed for the NCS or composite scores. *Discussion*. EZE/SIMV and ROSUV are superior to placebo in reducing LPO in subjects with T2DM suffering from polyneuropathy. This trial is registered with NCT02129231.

## 1. Introduction

Nerve damage in patients with diabetes is known as diabetic neuropathy and is considered as the most prevalent microvascular complication—up to 60%—in Type 2 Diabetes Mellitus (T2DM) subjects [1]. Diabetic polyneuropathy (DPN) comprises approximately 70% of all cases [2]. DPN diagnosis is established by means of validated scores based on clinical features and abnormal nerve conduction studies (NCS) [3]. Pathophysiologic findings include loss of multifocal and focal nerve fibers secondary to axonal degeneration and segmental demyelization. Damage to the nerves in diabetic subjects has been commonly associated with oxidative stress (OS) induced by chronic hyperglycemia [4–6]. One of the mechanisms proposed by which OS results in nervous system injury in diabetes suggests that high glucose increases the production of reactive oxygen species (ROS) and mitochondria damage, which precedes neuronal apoptosis [7]. Recent findings in type 1 diabetic patients (T1DM) have demonstrated a large benefit in the prevention of neuropathy from enhanced glucose control [8], whereas the benefit in T2DM is less evident [9, 10]. Thus, other mechanisms may contribute to the development of DPN in T2DM [11, 12]. Epidemiological studies have suggested that dyslipidemia is a risk factor for diabetic neuropathy [12, 13]. Likewise, experimental animal models have demonstrated a possible coregulation mechanism connecting hyperlipidemia and axonal degeneration [14].

The 3-hydroxy-3-methylglutaryl coenzyme A reductase inhibitors (statins) are potent inhibitors of cholesterol biosynthesis. Various clinical trials have shown beneficial effects of statins in the prevention of cardiovascular diseases [15]. However, pleiotropic effects of statins have also been suggested to occur through independent effects on lipid levels [16], such as neuroprotection in diabetic patients [17].

Simvastatin (SIMV), a lipophilic statin with intrinsic antioxidant activity, has been demonstrated to possess higher antihydroxyl radical activity than other statins [18]. Similarly, rosuvastatin (ROSUV), a hydrophilic statin, upregulates the antioxidant defenses and reduces NADPH-dependent production of oxygen radicals* in vitro* [19], as well as reducing OS in patients with dyslipidemia [20].

Thus, based on the aforementioned evidence regarding the plausible relationship between OS and DPN, and the promising pleiotropic effects of statins on this scenario, this randomized clinical trial was performed to evaluate the value of SIMV and ROSUV concerning the reduction of OS in patients with T2DM and DPN.

## 2. Methods

### 2.1. Study Design

A randomized, double-blind, placebo-controlled phase III clinical trial was performed at the Clinical and Experimental Therapeutics Institute, University of Guadalajara, Mexico. Subjects were assigned to three group treatments in blocks with a parallel sequence 1 : 1 : 1, through a randomized computer-based list generated by a different researcher unaware of the drugs given. Patients received once-a-day single-dose for 16 weeks of each treatment: controls received placebo, ezetimibe/SIMV (EZE/SIMV) 10/20 mg, and ROSUV 20 mg. We wanted to evaluate two high potency statins; in our Country, we lack rosuvastatin 5 mg dosage. Simvastatin monotherapy doses of 80 mg/day equal rosuvastatin 10 mg; however, high doses of statins increase the risk of adverse reactions; that is why we chose a combination therapy to reach similar effects and reduce adverse events. Patients were instructed to take their drugs in the evening at the same time every day. All drugs were similar in physical characteristics and presented in dark bottles, carefully filled by another group researcher who placed a respective tag with the patient code. Apart, patients were provided with a diary, whereby they wrote down the date and time of drug administration, as well as any drug adverse reactions experienced. Compliance was assessed by a coresearcher through pill counting and review of the diary provided. Such information was collected and registered every 4 weeks. The selection period was performed from February 2012 to January 2013. We did not influence or change their standard medications or lifestyle (dietary patterns and physical activity) during the study. Their family doctor was in charge of ensuring metabolic control, and we established frequent communication with them, to ensure none of the drugs implemented in our protocol suffered modifications. Also, patients were referred to their family physician or specialist if urgent treatment with statins or vitamin supplementation was required.

### 2.2. Study Population

Inclusion criteria were as follows: ≥18 years old, T2DM defined by the American Diabetes Association criteria, DPN defined by Dyck [3] criteria, HbA1c < 12%, and informed consent signed. Among others, exclusion criteria were renal or hepatic failure, pregnancy or breastfeeding, other neuropathies (alcohol-induced, radiculopathy, autoimmune, and cancer-related), lack of treatment adherence (<80% of drug intake), severe adverse drug reaction, and/or serious health illness. Subjects taking antioxidants, vitamins (B, C, and E), or statins up to three months previous to enrollment were also excluded. They were selected by invitation at forums, outpatients were recruited from primary care clinics, and database was collected previously by our institute from February 2010 to 2012. Primary outcomes were Lipid Peroxidation (LPO) and nitric oxide (NO) levels before and after 16 weeks of intervention. Secondary outcomes were nerve conduction studies (NCS), neuropathic symptoms score (NSS), neuropathic disability score (NDS), analog pain scale, and metabolic (fasting glucose, HbA1c, total cholesterol [TC], high and low density lipoproteins [HDL and LDL, resp.], and triglycerides) parameters. Safety profile was assessed by means of drug adverse reactions and renal (urea, creatinine) and hepatic (alanine and aspartate transaminase, gamma glutamyl transferase, bilirubin, and creatine kinase) laboratory variables.

### 2.3. LPO Assay

When polyunsaturated fatty acids are oxidized by ROS, malondialdehyde (MDA) is produced upon fatty acid decomposition; thus, measurement of MDA has been used as an indicator of LPO. LPO plasma levels were measured by a commercial kit (Oxford Biomedical Research Inc., FR12) according to manufacturer's instructions. This assay is based on the reaction of a chromogenic reagent, N-methyl-2-phenylindole, with MDA, which produces a chromophore with maximal absorbance at 586 nm. Results are expressed in *μ*M.

### 2.4. NO Levels

The plasma NO levels were indirectly estimated based on the determination of the NO metabolites, nitrate, and nitrite (NO_*x*_), according to manufacturer's instructions using a colorimetric assay kit (482650, Calbiochem). Plasma NO_*x*_ quantification is based on the Griess reaction. In brief, nitrate is converted to nitrite with cofactor and nitrate reductase, and then total nitrite reacts with the Griess reagent, thereby forming a deep purple azo compound which absorbs light at 540 nm. Results are expressed as pmol/mL.

### 2.5. Clinical and Nerve Conduction Variables

The NSS and NDS described by Dyck were obtained by physical examination and anamnesis [3]. We also measured the latency, duration, amplitude, and motor nerve conduction velocity from fibula, tibiae, median, and ulnae nerves and sensitivity parameters from sural, median, and ulnae nerves, as required by the American Association of Electrodiagnostic Medicine [21].

### 2.6. Ethical Considerations

The study was approved by the Research and Ethics Committee of the Health Science University Center, University of Guadalajara, Mexico, and by international instances (National Institutes of Health) with clinical trial identifier NCT02129231. Identification codes were assigned to each participant to guarantee patient confidentiality, and an informed consent form was signed before entering the protocol, according to national and international laws and also as stipulated by the Helsinki Statements (http://www.wma.net/es/30publications/10policies/b3/17c.pdf, accessed January 2011).

### 2.7. Statistical Analysis

The sample size was obtained by a clinical study design formula taking into account a difference change of 0.05 *μ*M in LPO, 95% confidence interval, 80% potency, and two-tailed *p* < 0.05, which resulted in 21 for each group. Quantitative variables were expressed as mean ± SD or SEM. Kolmogorov-Smirnov and Shapiro-Wilk tests were performed to determine the distribution of variables. Friedman and Wilcoxon tests were used before and after analysis, and Kruskal-Wallis with Mann-Whitney *U* as* post hoc* analysis between groups comparison. Qualitative variables were expressed as frequencies and percentages. McNemar test was used to evaluate differences in dichotomy variables before and after treatment and between groups comparison Fisher's exact and *χ* ^2^ tests were used as appropriate. Significance level was established with a *p* value <0.05.

## 3. Results

### 3.1. Baseline Clinical Characteristics

**Figure 1:**
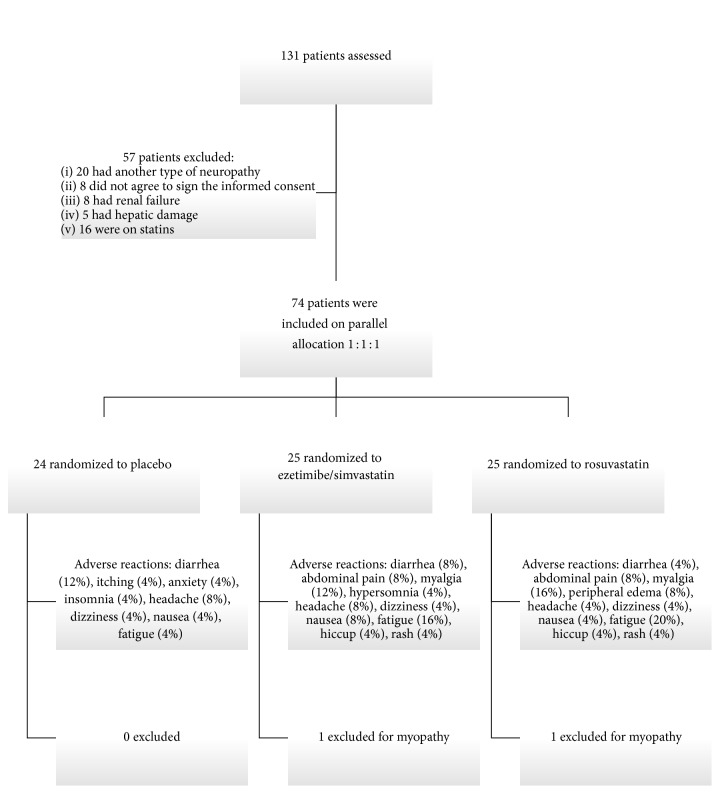
Flow diagram of study selection.

We assessed 131 patients, 57 were not eligible, and 74 were included and further divided into groups as follows: placebo, 24; EZE/SIMV, 25; and ROSUV, 25 (Figure 1). There were no significant differences on demographic characteristics at baseline between groups (Table 1). The mean age of the patients was 54.55 ± 1.2 years, 45 (60%) being women. The mean duration of T2DM was 10 years. All arm groups had high percentage of overweight and obese subjects.

### 3.2. Oxidative Stress Markers

**Figure 2:**
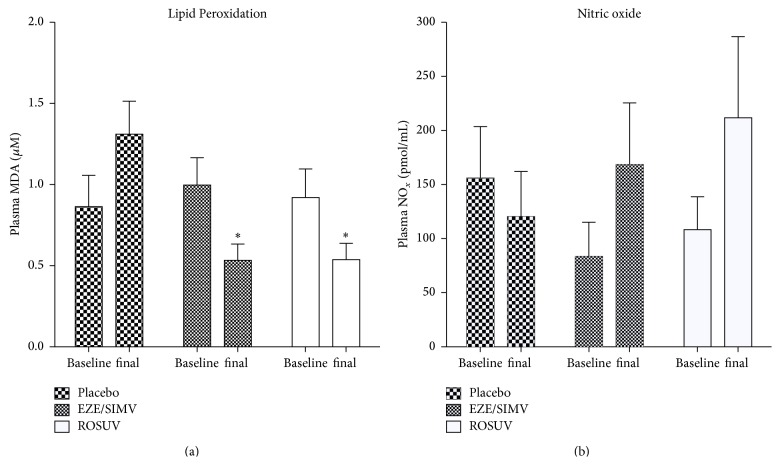
Oxidative stress (OS) and vascular function markers. (a) Lipid Peroxidation (LPO) levels in plasma, as assessed by malondialdehyde concentration. (b) Nitric oxide levels in plasma, as assessed by nitrate/nitrite (NO_*x*_) concentration. EZE/SIMV, ezetimibe/simvastatin; ROSUV, rosuvastatin. Data is expressed as mean ± SEM, ^*∗*^ *p* < 0.05 versus placebo Mann-Whitney *U*.

Basal LPO levels in the placebo, EZE/SIMV, and ROSUV groups were 0.92 ± 0.20, 0.99 ± 0.15, and 0.82 ± 0.15 *μ*M, respectively (Figure 2(a)). No significant differences between groups were observed at baseline (*p* = 0.253, Kruskal-Wallis). After 16 weeks, placebo group showed increased LPO levels up to 1.31 ± 0.20 *μ*M (*p* < 0.05 baseline versus final) (Figure 2(a)). On the other hand, EZE/SIMV and ROSUV groups significantly improved LPO levels to 0.52 ± 0.10 *μ*M (*p* < 0.05 baseline versus final) and 0.53 ± 0.10 *μ*M (*p* < 0.05 baseline versus final), respectively (Figure 2(a)).

### 3.3. NO Levels

Basal NO levels in the placebo, EZE/SIMV, and ROSUV groups were 156.0 ± 47.58, 83.43 ± 31.72, and 108.3 ± 30.36 pmol/mL, respectively (Figure 2(b)). At baseline, no significant differences between groups were found (*p* = 0.15, Kruskal-Wallis). After 16 weeks, placebo group showed 120.25 ± 41.8 pmol/mL (*p* = 0.511 baseline versus final), EZE/SIMV 168.57 ± 54.13 pmol/mL (*p* = 0.360 baseline versus final), and ROSUV 211.73 ± 75.05 pmol/mL (*p* = 0.814 baseline versus final) NO levels.

### 3.4. Clinical Outcomes and NCS

**Figure 3:**
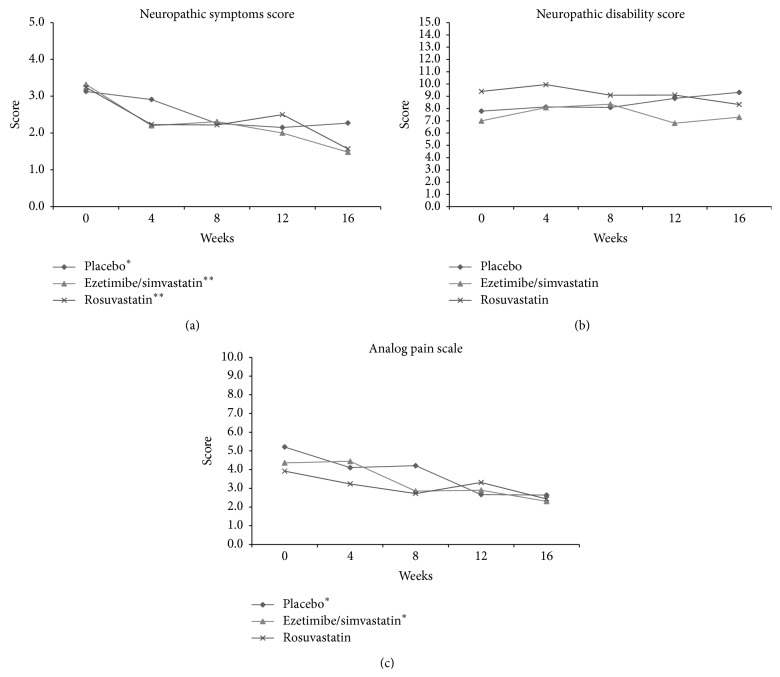
Screening levels and changes from screening in (a) neuropathic symptoms score (NSC), (b) neuropathic disability score (NDS), and (c) analog pains scale (APS) score after 16 weeks of treatment. Data is expressed as mean ± SEM, ^*∗*^ *p* < 0.05 versus week 0, and ^*∗∗*^ *p* < 0.001 versus week 0 (baseline), Wilcoxon matched-pairs signed-rank test.

Baseline NSS values in the placebo, EZE/SIMV, and ROSUV groups were 3.1 ± 1.8, 3.3 ± 1.8, and 3.2 ± 1.9 (*p* = 0.851 between groups), respectively. We observed significant reductions in the placebo group to 2.2 ± 1.6 (*p* < 0.01 baseline versus final), EZE/SIMV 1.4 ± 1.5 (*p* < 0.001 baseline versus final), and ROSUV 1.5 ± 1.4 (*p* < 0.01 baseline versus final). Noteworthy, the EZE/SIMV and ROSUV groups showed greater improvement than placebo group (Figure 3(a)). At baseline, NDS were 7.7 ± 5.8, 7.0 ± 4.6, and 9.4 ± 5.1 for placebo, EZE/SIMV, and ROSUV, respectively (*p* = 0.110 between groups) (Figure 3(b)). At the end of the intervention, there were no significant differences on NDS in the placebo (*p* = 0.716 baseline versus final), EZE/SIMV (*p* = 0.834 baseline versus final), and ROSUV (*p* = 0.432 baseline versus final) groups. Basal analogue pain scale (APS) values were 5.2 ± 0.7, 4.4 ± 0.8, and 3.9 ± 0.7 (*p* = 0.463 between groups), in the placebo, EZE/SIMV, and ROSUV groups, respectively. We observed a reduction on APS values of 2.6 ± 0.0 (*p* = 0.003 baseline versus final), 2.1 ± 0.0 (*p* = 0.006 baseline versus final), and 1.5 ± 0.1 (*p* < 0.095 baseline versus final) in the placebo, EZE/SIMV, and ROSUV groups, respectively (Figure 3(c)).

The electrophysiological data are shown in Table 2. At baseline NCS were similar in all groups, with no significant differences between groups. Placebo group exhibited a reduction of 0.4 ms on the sural nerve latency (*p* < 0.01 baseline versus final) and an increase of 1.3 and 1.0 m/s on the peroneal and tibiae nerve velocities, respectively (*p* < 0.01 baseline versus final). In the EZE/SIMV group there were a reduction of 0.4 ms on the sural nerve latency (*p* < 0.05 baseline versus final), an increase of 1.5 mV on the median motor nerve amplitude (*p* = NS baseline versus final), and an increase of 0.4 mV on the median sensitive nerve latency (*p* = NS baseline versus final).

### 3.5. Metabolic and Safety Profile Parameters

Metabolic characteristics are shown in Table 3. Baseline metabolic variables were heterogeneous, with differences in fasting glycaemia between EZE/SIMV compared to ROSUV arm (*p* = 0.013), HbA1c (*p* = 0.044), and total bilirubin (*p* = 0.003). At the end of the study, there was a reduction in fasting plasma glucose in the placebo group (*p* = 0.004) and LDL (*p* = 0.01). In the EZE/SIMV group there were a significant reduction on TC 82.8 ± 49.8 mg/dL (*p* = 0.001) and LDL 57.1 ± 48.4 mg/dL (*p* = 0.001) and a trend towards significance on TG levels (*p* = 0.055). In the ROSUV arm CT, LDL, and TG were reduced by 73.3 ± 49.5 mg/dL (*p* = 0.001), 64.9 ± 44.0 mg/dL (*p* = 0.001), and 41.1 ± 61.2 mg/dL (*p* = 0.003), respectively. Also, a significant reduction was observed concerning bilirubin levels (*p* = 0.02). We also report on gastrointestinal, neurologic, dermatologic, and muscular adverse drug reactions, and 2 patients were eliminated due to statin-related myopathy (one in each group) (see Figure 1).

## 4. Discussion

Diabetes can damage the peripheral nervous system in various ways, DPN being the most common presentation [22]. DPN is one of the major complications of DM leading to an increased rate of morbidity and mortality among diabetic patients [23, 24]. The precise mechanisms of this pathology remain elusive, and few interventions are available to alleviate the nonpainful symptoms. Thus far, glucose control is the only proven disease-modifying intervention available for diabetic subjects suffering from DPN. However, despite the robust effect that glucose control has on neuropathy in T1DM subjects, this effect is much smaller in T2DM [22]. Therefore, it has been suggested that other modifiable risk factors for neuropathy may play a more relevant role in T2DM subjects. Noteworthy, the incidence of dyslipidemia is high in T2DM [25], and this homeostasis imbalance of lipids has been correlated with the progression of diabetic neuropathy [12]. In a cross-sectional and longitudinal analysis of the Fremantle Diabetes Study the data suggest that therapy with statin or a fibrate may protect against DPN in T2DM subjects [17]. One randomized clinical study evaluated the effects of statins in diabetic neuropathy, whereby the authors suggested a relative small benefit on nerve conduction velocity parameters after six-month statin therapy in noninsulin dependent diabetic subjects [26].

There are several underlying mechanisms suggested to be linked to the development and progression of DPN caused by dyslipidemias [11]. A convergent point for such mechanisms is thought to be OS, which is suggested to be responsible for the pathophysiologic changes observed in T2DM subjects that leads to axonal degeneration and segmental demyelination, thereby promoting DPN [2–4]. LPO has been frequently associated with OS in human diseases and is commonly used as biomarker of OS [27, 28]. Moreover, LPO of nerve membranes has been proposed to lead to peripheral nerve ischemia and hypoxia, which in turn may contribute to the development of neuropathy. Hence, the prevention and/or improvement of DPN by means of OS reduction are in current investigation. Here, our data shows that statin therapy with both EZE/SIMV and ROSUV was more effective than placebo in reducing plasma LPO levels. Interestingly, in support of our findings Koksal et al. suggested that 10 mg/day ROSUV for 3 months may be helpful in reducing the increased OS observed in T2DM subjects with hyperlipidemia [29]. Similarly, Yoshino et al. suggested that 2.5 mg/day ROSUV for 3 months was associated with a reduction in plasma and urine OS markers in hypercholesterolemic patients [30]. Nonetheless, the aforementioned studies lack a placebo group, which was included in our study. Likewise, Girona et al. demonstrated that SIMV possesses the ability to decrease aldehyde production derived from lipoprotein peroxidation in humans [31]. Thus, this study clearly evidences that statin therapy may reduce cellular injury caused by OS in T2DM subjects with DPN by means of its capacity to decrease LPO, corroborating other studies.

Furthermore, as patients with diabetes exhibit impaired NO availability, thereby contributing to endothelial dysfunction [32], we evaluated the NO levels in these patients in our study. Although we did not observe a significant effect on NO levels in both statin groups, there was an increase of approximately 95% in NO levels on both statin arms, while in the placebo group there was a reduction of approximately 20%. This increase may be partially related with the decrease of the oxidant environment (i.e., LPO levels), suggesting a beneficial effect of statins in the vasculature, as shown by others [33–35].

Although OS has been implicated in the development and progression of diabetic neuropathies, we did not observe a superior effect of statins over placebo on the clinical outcomes after 16 weeks of intervention. However, we observed a trend towards significance on the NSS values in the EZE/SIMV group when compared to placebo (*p* = 0.052), which may suggest that a lengthened treatment is necessary to achieve a significant difference. As expected, the lipid profile was favorably affected (i.e., reduction on TC, LDL, and TG) by the statin treatment when compared to placebo. Unexpectedly, the placebo group improved fasting plasma glucose, without repercussion on HBA1C. Regarding safety issues, we did not observe any significant elevation neither on liver enzymes nor creatinine by treatment with statins.

Limitations include the lack of homogenization of lifestyle changes previous to randomization and throughout the study. Some of the patients could change their antidiabetic medications during the protocol, because their family physician was in charge of their glucose control; however, we ensured no statins and/or antioxidants were taken during the duration of the study. Most of the clinical trials that evaluate DPN are performed for a minimum of 12 months to ensure modifications in clinical outcomes; probably the duration of our trial was too short to demonstrate changes in clinical and nerve conduction parameters; however, the main objective was to evaluate the statins effect on oxidative stress.

In summary, this trial demonstrated that EZE/SIMV and ROSUV are superior to placebo in reducing LPO levels in T2DM after 16 weeks of treatment. Future larger randomized clinical trials and for longer period of time are needed, in order to confirm the favorable effects that statins may have on OS in T2DM subjects suffering from DPN.

## Figures and Tables

**Table 1:** Clinical characteristics. Different population variables of each study group are enlisted; none of them were statistically different between groups.

Clinical characteristics	Placebo *n* = 24	EZE/SIMV *n* = 25	ROSUV *n* = 25
Gender (M/F)^†^, *n* (%)	7/17 (29/71)	10/15 (40/60)	12/13 (48/52)

Age (years)^*∗*^	54.7 ± 9.6	55.0 ± 12.0	54.0 ± 10.5

Weight (kg)^*∗*^	73.7 ± 11.4	75.4 ± 13.9	76.9 ± 18.7

Height (mt)^*∗*^	1.59 ± 0.09	1.60 ± 0.10	1.62 ± 0.13

Body mass index (kg/m^2^)^*∗*^	29.3 ± 4.3	29.4 ± 4.1	29.0 ± 4.7

DM type 2 duration (years)^†^	10.5 ± 8.3	10.2 ± 6.6	12.1 ± 8.3

Systolic blood pressure (mmHg)^*∗*^	142 ± 25	144 ± 25	135 ± 17

Diastolic blood pressure (mmHg)^*∗*^	84 ± 11	81 ± 10	81 ± 7

Smoking (Y/N)^†^, *n* (%)	9/15 (38/62)	8/17 (32/68)	12/13 (48/52)

Concomitant drugs, *n* (%)^†^			
NSAID	1 (4)	2 (8)	2 (8)
Angiotensin Converter Enzyme Inhibitor	3 (13)	3 (13)	2 (8)
Angiotensin Receptor Blocker	2 (8)	1 (4)	
Calcium antagonist	2 (8)		
Aspirin	1 (4)		1 (4)
Proton Pump Inhibitors		2 (8)	2 (8)
Benzodiazepine	1 (4)		
Diuretics		1 (4)	3 (13)

DM type 2 treatment, *n* (%)			
Insulin	1 (4.2)	2 (8.0)	4 (16.0)
Metformin	5 (20.8)	5 (20.0)	3 (12.0)
Glyburide	15 (62.5)	1 (4.0)	11 (44.0)
Metformin/glyburide	1 (4.2)	13 (52.0)	2 (8.0)
Metformin/insulin	2 (8.3)	2 (8.0)	3 (12.0)
Metformin/glyburide/insulin		2 (8.0)	2 (8.0)
Other combinations			

Mean ± SD unless specified different. ^*∗*^ *p* = NS Kruskal-Wallis, ^†^ *p* = NS. DM, diabetes mellitus; NSAID, nonsteroid anti-inflammatory drug.

**Table 2:** Nerve conduction studies. Values of different nerve parameters are reported before and after treatment in all groups.

	PLACEBO			EZE/SIMV			ROSUV		
	*n* = 24			*n* = 25			*n* = 25		
	Baseline	Final	*p*	Baseline	Final	*p*	Baseline	Final	*p*
Lower limbs									
Sural nerve									
Lat (ms)	4.0 ± 0.5	3.6 ± 0.2	**0.006**	4.1 ± 0.4	3.7 ± 0.1	**0.021**	4.1 ± 0.6	3.8 ± 0.2	0.070
Amp (*µ*V)	10.9 ± 12.0	15.9 ± 2.8	0.084	9.5 ± 3.2	14.8 ± 2.9	0.477	12.6 ± 6.6	15.2 ± 2.3	0.538
Peroneal nerve									
Lat (ms)	3.8 ± 0.8	3.7 ± 0.2	0.207	3.7 ± 0.5	3.6 ± 0.2	0.210	3.6 ± 0.9	3.5 ± 0.1	0.810
Amp (mV)	4.7 ± 3.1	5.0 ± 0.5	0.191	4.5 ± 1.7	4.4 ± 0.4	0.835	4.7 ± 2.6	4.5 ± 0.5	0.296
Vel (m/s)	39.3 ± 4.4	40.6 ± 1.1	**0.004**	40.6 ± 3.8	41.5 ± 0.9	0.111	39.9 ± 4.1	40.0 ± 0.8	0.944
Tibiae nerve									
Lat (ms)	4.2 ± 1.3	4.1 ± 0.2	0.395	4.4 ± 0.9	4.5 ± 0.3	0.562	4.3 ± 1.1	4.3 ± 0.2	0.776
Amp (mV)	8.4 ± 4.3	9.1 ± 1.2	0.070	8.3 ± 3.3	7.9 ± 0.7	0.390	8.2 ± 3.2	8.3 ± 0.9	0.972
Vel (m/s)	42.1 ± 5.4	43.1 ± 1.3	**0.008**	44.2 ± 5.2	45.0 ± 0.9	0.057	45.2 ± 6.3	44.4 ± 1.3	0.136
Upper limbs									
Median motor nerve									
Lat (ms)	4.6 ± 1.4	5.5 ± 0.5	0.655	6.5 ± 1.8	5.6 ± 1.0	0.655	4.4 ± 0.8	5.0 ± 0.1	0.317
Amp (mV)	6.0 ± 1.3	4.5 ± 0.9	0.180	6.5 ± 1.0	8.0 ± 0.8	0.655	9.2 ± 3.9	7.3 ± 1.4	0.180
Vel (m/s)	47.4 ± 5.9	45.7 ± 3.8	0.180	49.7 ± 3.8	51.2 ± 2.3	1.000	41.7 ± 3.5	51.3 ± 4.9	0.317
Median sensitive nerve									
Lat (ms)	3.8 ± 0.6	3.7 ± 0.1	0.141	3.9 ± 0.6	**4.3 ± 0.2** ^†^	0.076	4.3 ± 0.7	**4.2 ± 0.2** ^**∗**^	0.539
Amp (*µ*V)	26.0 ± 14.5	26.4 ± 4.0	0.553	22.2 ± 14.0	18.3 ± 2.4	0.476	15.8 ± 6.4	21.6 ± 2.5	0.266
Vel (m/s)	52.2 ± 6.0	53.1 ± 1.3	0.195	51.1 ± 5.2	52.8 ± 1.3	0.083	52.8 ± 5.2	52.8 ± 1.5	0.778
Ulnar motor nerve									
Lat (ms)	3.5 ± 0.6	3.2 ± 0.1	0.031	3.3 ± 0.3	3.4 ± 0.1	0.479	3.4 ± 0.4	3.3 ± 0.1	0.468
Amp (mV)	8.3 ± 2.7	8.2 ± 0.7	0.888	8.9 ± 2.7	8.0 ± 0.3	0.338	8.3 ± 1.6	8.1 ± 0.4	0.394
Vel (m/s)	51.2 ± 8.5	51.6 ± 2.0	0.776	55.0 ± 4.8	53.7 ± 1.3	0.198	54.0 ± 4.4	53.9 ± 1.1	0.649
Ulnar sensitive nerve									
Lat (ms)	3.8 ± 0.8	3.6 ± 0.1	0.629	3.7 ± 0.4	3.8 ± 0.1	0.673	3.7 ± 0.7	3.7 ± 0.1	0.175
Amp (*µ*V)	21.2 ± 23.9	21.5 ± 2.9	0.127	19.8 ± 12.6	19.5 ± 3.1	0.936	16.9 ± 7.7	17.8 ± 2.2	0.913
Vel (m/s)	53.7 ± 4.5	53.5 ± 1.5	0.463	55.5 ± 5.7	55.1 ± 1.2	0.380	54.3 ± 5.7	54.3 ± 1.1	0.448

Mean ± SEM, ^*∗*^ *p* < 0.05, ^†^ *p* < 0.01 versus placebo Mann-Whitney's *U*.

**Table 3:** Metabolic characteristics. Biochemical parameters are reported for each treatment group before and after intervention.

	Placebo			Ezetimibe/simvastatin			Rosuvastatin		
	*n* = 24			*n* = 25			*n* = 25		
	Baseline	Final	*p*	Baseline	Final	*p*	Baseline	Final	*p*
Fasting plasma glucose (mg/dL)	186.4 ± 14.48	150.2 ± 11.20	**0.004**	146.64 ± 9.92	152.6 ± 14.00	0.620	192.04 ± 12.73	179.2 ± 15.20	0.520
Urea (mg/dL)	29.5 ± 2.1	28.9 ± 2.2	0.520	30.03 ± 1.51	32.5 ± 1.9	0.280	31.22 ± 2.27	28.6 ± 2.2	0.300
Creatinine (mg/dL)	0.79 ± 0.04	0.84 ± 0.05	0.180	0.82 ± 0.04	0.85 ± 0.05	0.570	0.84 ± 0.04	0.80 ± 0.04	0.320
AST (U/L)	21.9 ± 1.89	21.8 ± 1.4	0.820	24.63 ± 0.99	27.1 ± 2.5^*∗*^	0.270	27.8 ± 1.91	26.7 ± 1.2^∧^	0.700
ALT (U/L)	20.2 ± 1.43	20.5 ± 1.9	0.990	28.57 ± 3.26	28.4 ± 3.1^*∗*^	0.570	31.22 ± 2.47	30.3 ± 2.4^∧^	0.870
GGT (U/L)	34.73 ± 6.49	39.4 ± 8.6	0.270	39.83 ± 9.04	33.7 ± 3.7	0.780	44.33 ± 6.93	43.9 ± 7.7	0.260
Bilirubin (mg/dL)	0.70 ± 0.06	0.56 ± 0.05	0.052	0.61 ± 0.04	0.64 ± 0.06	0.800	0.83 ± 0.05	0.65 ± 0.07	**0.020**
Cholesterol (mg/dL)	211.43 ± 11.73	202.3 ± 8	0.610	210.56 ± 9.94	129.3 ± 9.7^*¥*^	**0.001**	217.2 ± 8.04	142.7 ± 8.8^*¥*^	**0.001**
LDL (mg/dL)	126.68 ± 8.76	109.6 ± 7.8	**0.010**	117.45 ± 7.16	61.7 ± 6.1^*¥*^	**0.001**	133.56 ± 7.96	75 ± 8.1^*¥*^	**0.001**
HDL (mg/dL)	36.95 ± 2.61	39.7 ± 2.5	0.470	36.23 ± 2.06	32.7 ± 2.1	0.090	36.79 ± 1.91	36.5 ± 2.3	0.360
Triglycerides (mg/dL)	240.13 ± 27.41	242.4	0.590	234.63 ± 49.96	161.9 ± 21.2^*∗*^	0.055	220.6 ± 24.49	168.4 ± 24.4^*∗*^	**0.003**
CK (U/L)	82.21 ± 13.78	82.8 ± 10.3	0.930	95.92 ± 14.4	86.4 ± 8.3	0.460	114.93 ± 18.44	157.3 ± 35.1	0.190
HBA1C % (mmol/mol)	8.8 (73) ± 0.36	9.2 (77) ± 0.5	0.260	7.8 (62) ± 0.32	8.1 (65) ± 0.4	0.250	9.0 (75) ± 0.40	9.4 (79) ± 0.4^*∞*^	0.090

Mean ± SEM ^*∗*^ *p* < 0.05  versus placebo; ^∧^ *p* < 0.01  versus placebo; ^*¥*^ *p* < 0.001 versus placebo; ^*∞*^ *p* < 0.05 versus ezetimibe/simvastatin (Mann-Whitney's *U*). ALT, alanine transaminase; AST, aspartate transaminase; CPK, creatine kinase; HBA1C, glycated hemoglobin; HDL, high-density lipoproteins; GGT, gamma glutamyl transferase; LDL, low-density lipoproteins.
